# Atomic force microscopy of chromatin arrays reveal non-monotonic salt dependence of array compaction in solution

**DOI:** 10.1371/journal.pone.0173459

**Published:** 2017-03-15

**Authors:** Katarzyna M. Krzemien, Maximilian Beckers, Salina Quack, Jens Michaelis

**Affiliations:** Institute of Biophysics, Ulm University, Ulm, Germany; National Cancer Institute, UNITED STATES

## Abstract

Compaction of DNA in chromatin is a hallmark of the eukaryotic cell and unravelling its structure is required for an understanding of DNA involving processes. Despite strong experimental efforts, many questions concerning the DNA packing are open. In particular, it is heavily debated whether an ordered structure referred to as the “30 nm fibre” exist *in vivo*. Scanning probe microscopy has become a cutting edge technology for the high-resolution imaging of DNA- protein complexes. Here, we perform high-resolution atomic force microscopy of non-cross-linked chromatin arrays in liquid, under different salt conditions. A statistical analysis of the data reveals that array compaction is salt dependent in a non-monotonic fashion. A simple physical model can qualitatively explain the observed findings due to the opposing effects of salt dependent stiffening of DNA, nucleosome stability and histone-histone interactions. While for different salt concentrations different compaction states are observed, our data do not provide support for the existence of regular chromatin fibres. Our studies add new insights into chromatin structure, and with that contribute to a further understanding of the DNA condensation.

## Introduction

Chromatin is the solution of the eukaryotic cell to fulfil two opposing tasks: a high compaction of DNA and fast and controlled access to the genomic information. The basic repeating unit in chromatin is the nucleosome, in which 146 bp of DNA are wrapped around a protein core, the histone octamer [1]. The histone octamer is a disc-shaped protein complex containing two copies of each of the four histone proteins (H2A, H2B, H3, and H4), were the H3-H4 tetramer binds the inner ≈56 bp of DNA, and H2A-H2B dimers are situated at either side binding to 27-28 bps of DNA [2]. Since, *in vivo*, large fractions of DNA (e.g. ≈ 80% in yeast genome [3]) are bound to histones, nucleosomes are connected by a linker DNA, creating structures called “beads on a string”. The length of linker DNA is varying between species and different tissues in a range from 0 to 80 bp [4], and linker lengths are occurring in multiple of ≈10 bps caused by the helicity of the DNA [5]. While the compaction into nucleosomes is well accepted, the next stage of nucleosome compaction into so called chromatin fibres is unclear. There are publications presenting proofs for highly organised chromatin structure *in vitro* using electron microscopy (EM) to visualise G1 chromosomes [6] or in long reconstructed chromatin arrays [7, 8]. Also modelling studies [9, 10] support the existence of highly organised chromatin structures and propose different, well ordered structural models for the chromatin fibre. On the other hand side, other groups using EM together with small angle X- ray scattering (SAXS) [11], electron spectroscopic imaging (ESI) together with tomography [12], Hi-C-based method called Micro-C [13] and stochastic optical reconstruction microscopy (STORM) [14] find no evidence for the existence of chromatin fibres *in vivo*, nor *in vitro* in recent studies combining fluorescence light (FM) and transmission electron (TEM) microscopy with sedimentation velocity analytical ultra-centrifugation (SV-AUC) and SAXS [15]. The scientific discussion around this controversial topic is summarised in several reviews in the last years [16–21]. Despite this controversy, providing answers about chromatin structure is indispensable for the complete understanding of basic cellular processes involving DNA, such as transcription, replication, recombination and maintaining the integrity of the genome.

By now, it has become clear that the structure of chromatin and its stability and variability is a complex problem, influenced by several factors. It has been shown using Atomic Force Microscopy (AFM) that post-translation modifications (PTM) have an effect on nucleosomes dynamic, especially an acetylation of chromatin studied on nucleosomal arrays [22]. *In vitro* single molecule magnetic tweezers and Förster Resonance Energy Transfer (FRET) studies proved, that the acetylation of histone H3 has different influence on the nucleosome stability and structure than the H4 acetylation [23, 24]. Also different PTM introduced in the same place within a nucleosome can define two opposing states of chromatin [25]. Additionally, the octamer composition, namely the presence of different histone variants, has an impact on the nucleosome stability [26, 27]. Recently, also the influence of DNA super-coiling on nucleosomal stability was investigated using AFM and Fluorescent Correlation Spectroscopy (FCS) [28]. Moreover, to test the stability of individual nucleosomes, the influence of salt on nucleosomes was studied using single molecule FRET (smFRET) [29], a combination of smFRET and FCS [30] and AFM [31]. Furthermore, chromatin purified from different cells was investigated under different cation concentrations by a large variety of methods, e.g. analytical ultra-centrifugation (AUC) and EM [32], EM, X-ray scattering and AUC [33], electric dichroism [34] and TEM together with AFM [35].

Thanks to developments in recombinant biochemistry methods, well-defined recombinant nucleosomal arrays became a model for studying chromatin compaction and its functional dependence on salt. Many of those studies in the past decades investigated the influence of magnesium ions using AUC [36–38]. In more recent studies, magnesium-induced self-assembly of nucleosomal arrays into globular oligomers was studied using a broader range of techniques (FM, TEM, SV-AUC and SAXS) [15]. Other studies considered potassium ions [39], as well as mixtures of potassium and magnesium or sodium and magnesium ions [40]. There are also studies on chromatin arrays employing solely sodium chloride, which were performed using AUC [41, 42]. These studies highlighted an increase in the sedimentation coefficients for increasing sodium concentrations. This would suggest a monotonic influence of sodium chloride on chromatin arrays, in contrast to the non-monotonic dependence of mononucleosomes on NaCl reported in recent single molecule studies [29]. This discrepancy motivates the need of more direct and systematic study of the compaction of chromatin arrays under different NaCl concentrations.

High-speed AFM, due to multiple technical improvements, has become very popular for studding DNA-protein complexes, recently reviewed by in [43]. Typically, the high-resolution AFM images of mono-nucleosomes and arrays were performed in air [22, 31, 44, 45] or, if in liquid, with the use of glutaraldehyde for fixation [35, 46, 47]. Additionally, Wang et al. used glutaraldehyde functionalised mica in order to immobilise the nucleosomal arrays [46]. Although they claim, that the nucleosomes tethered by glutaraldehyde are still mobile in arrays, they show that the histone protein remains fixed in place, while the DNA is free to move and eventually diffuses away from imaged arrays. An interesting alternative to chemical fixation is using mica covered with poly-L-lysine, which has been successfully used to image DNA by AFM [48]. The poly-L-lysine binds the DNA molecule by electrostatic interactions, with the strength dependent on the degree of polymerisation [49]. Poly-L-lysine coated surfaces were used to immobilise nucleosomes, both in air as well as in liquid [50], and for imaging chromatin arrays in air [23]. However, early trials to image full arrays in liquid without chemical cross-linking resulted in comparatively low-resolution images, as compared to the high-resolution scans performed in air [51, 52].

While a wealth of information has been obtained from various AFM experiments of nucleosomal arrays [22, 35, 45–47, 51–55], in order to gain mechanistic insight into salt dependent array compaction a good control of experimental conditions is important. This means that experiments need to be performed using well established array reconstitution protocols, where one can ensure a complete occupancy of the array. Moreover, one also needs to ensure a uniform chemical composition. This is not possible if complete arrays or histone proteins are purified from cells due to the varying concentration and composition of linker histones as well as the unknown post-translational modification state of the histone proteins. Thus, an assembly using recombinant proteins and a quality control for complete array occupancy is required.

Here, we present high-resolution AFM images of non-cross-linked, chromatin arrays in liquid. The amount of collected data together with a rigorous automated measurement tool followed by a statistical analysis allowed us to study the influence of sodium chloride concentrations on the compaction of nucleosomal arrays. Our results show that even small changes in the amount of sodium ions, lead to changes in compaction of the chromatin arrays. The NaCl influence on the arrays is non-monotonic, but simple energetic arguments can be used to explain the observed phenomena. Interestingly, we never observe a highly organised structure of chromatin arrays that would support the existence of 30 nm chromatin fibres.

## Materials and methods

### Histones expression and purification

Histones expression and purification followed largely the protocol described in Ref. [56]. In brief, the pET21 expression plasmids containing human histones sequences were a kind gift from G.Längst (University of Regensburg). Recombinant human histones were expressed in *E.coli* (BL21(DE3)) using standard LB medium containing 1 mM ampicillin. The cells were induced with 1 mM IPTG at OD_600_ between 0.55 and 0.65. Cultures for expressing H4 histone had 1.5-2 times larger volumes, due to lower expression yields [57]. After harvesting the cells, the pellets were shock frozen in liquid nitrogen and stored in -80°C until purification. For purification, the pellets were re-suspended directly in 50 mL of buffer SAU-200 (8 M urea, 40 mM sodium acetate pH 5.2, 1 mM EDTA pH 8, 200 mM NaCl, 10 mM L-lysine and 5 mM *β*-mercaptoethanol) with 1 mM PMSF added. Cell lysis was performed by homogenization in Potter-Elvehjem-type tissue grinder, followed by 4 minutes of effective sonication time (Digital Sonifier Cell Disruptor, Branson) at 30% amplitude and with 15 sec pulse ON and 30 sec pulse OFF. Afterwards, solution filtration was performed in three steps: 1) “cheese cloth” filtration through single layer of Miracloth (Calbiochem); 2) pre-filtration with syringe filter FP 30/50 CN 5 *μ*m (Whatmann, GE Healthcare); 3) filtration with PVDF 0.45 *μ*m syringe filters (Whatmann, GE Healthcare). Subsequently, cation exchange was implemented, using a tandem of two 1 mL HiTrap SP FF (GE Healthcare) columns. Gradient profile was performed as follows: 1) 200 mM NaCl, 4 CV; 2) 250 mM NaCl, 4 CV; 3) gradient from 250 mM to 600 mM NaCl for 8 CV; 4) gradient from 600 mM to 1 M NaCl for 4 CV; 5) 1 M, NaCl 1 CV. The fractions from each ion exchange run were run on a SDS-PAGE gel. Selected fractions were collected and dialysed over night against cold water (4°C) containing 1 mM DTT. The histones were then centrifuged (20 min., ≈38000rcf, 4°C), and filtered with a PVDF 0.45 *μ*m syringe filter. Finally, after adding Tris to obtain a final concentration of 15 mM, the histones were purified using anion exchange chromatography (HiTrap Q HP 1 mL; GE Healthcare Life Sciences). This last purification step is performed to separate histones from bound DNA strands. The DNA in 15 mM Tris buffer binds to the anion exchange column, while the purified proteins are found in the flow-trough fraction. For the recovery from the column, a linear gradient from 0 M to 1 M NaCl over 5 CV was used. The histones purified via this protocol were then aliquotted (1 mg per aliquot), freeze-dried (Alpha 1-2 LDplus, Martin Christ GmbH) and stored in -80°C for later use.

### Histone octamer reconstitution

The histone octamers were reconstituted from bacterially expressed human histones following the protocol established by Luger et al. [58]. Small modifications to the protocol were implemented according to Ref. [59].

### DNA preparation

For the reconstitution of chromatin arrays we used synthetic DNA containing 25 mers of the 197 bp ‘601’ nucleosome positioning sequence (later refereed to as 25 x ‘601’) [60]. The pUC18 plasmid containing this sequence was a kind gift from D. Rhodes (NTU, Singapore). XL1Blue and StbL3 *E.coli* cells were used to amplify DNA constructs. The amplification of plasmids using the StbL3 cell line, although often yielding lower DNA amounts as compared to expression in the XL1Blue cells, led to a lower mutation rate, ultimately making the StbL3 cell strain a preferable choice. The amplified DNA was digested with restriction enzymes HindIII-HF, EcoRI-HF and DraI (all from NEB) in a single pot reaction following the manufacturer protocol, in order to excise the insert with the 25 x ‘601’ DNA and to fragment the backbone of the plasmid (used as a competitor DNA in the array assembly). Afterwards, the DNA was purified using standard organic solvent extraction and ethanol precipitation protocols. The quality of amplified and digested DNA was confirmed by agarose gel electrophoresis.

### Chromatin arrays assembly

The chromatin arrays were assembled from 25 x ‘601’ DNA and recombinant human histones, following largely the salt gradient dialysis protocol from Ref. [59]. To optimize the loading of the DNA with histone octamers, a titration series was employed [61]. 5 mg of 25 x ‘601’ DNA were used per titration. The DraI fragmented backbone of the plasmid served as a low affinity competitor DNA. The DNA (total amount ≈7.7 mg) was combined with varying amounts of histone octamers (titration ratios of 1:0.7 to 1:2.8) in a buffer containing 10 mM Tris and 2 M NaCl in a total volume of 50 *μ*L. Each titration was pipetted into a Slide-A-Lyzer^™^ MINI Dialysis Device and dialysed against buffer containing 10 mM Tris pH 7.5, 0.01% NP40, 1 mM DTT and decreasing salt gradient from 2 M NaCl to a final concentration of 50 mM NaCl (≈24 h, 4°C).

For purification of the assembled arrays precipitation with MgCl_2_ was performed [36]. Volume and concentration of each sample were measured. Afterwards, a centrifugation step was performed (15 min., 20800 rcf, 4°C), the supernatant of each sample was transferred to new low binding eppendorf tube and the concentration of each array was measured again. Samples that underwent a significant concentration loss were discarded. An equivalent volume containing 8 mM MgCl_2_ was added to each sample. The mixture was then incubated for 15 minutes on ice. After another centrifugation step (15 min., 20800 rcf, 4°C) the supernatant was discarded and the pellets were re-suspended in 10 mM Tris to a final concentration of 300 ng/*μ*L. The arrays in this concentration were stored in 4°C, and were stable for about a couple of months. As quality control of the arrays, native agarose gel of samples from different stages of MgCl_2_ precipitation was used, as well as AvaI and AluI restriction digestion tests, as described in [59]. The AvaI restriction site is located in the 50 bp linker DNA. Conversely, the AluI restriction site is located in the middle of the ‘601’ positioning sequence. An array properly saturated with histone octamers has none of the AluI restriction sites available and all AvaI sides exposed to the enzyme. The under-saturated array has some of the AluI restriction side accessible for the endonuclease. In case when the DNA is over-saturated with octamers, some of the AvaI digestion sites are not accessible for the enzyme, resulting in several bands of length exceeding 200 bp. Additionally, if the the excess of protein complex on DNA strand is significant, the octamers will be considerably moved away from the original positioning sequence, exposing AluI restriction sites and showing additional digestion patterns also in the AluI digestion test.

### Sample preparation and AFM measurements

160 *μ*L of poly-L-lysine (MW 150,000-300,000, Sigma Aldrich) water solution (0.01%) were deposited on a freshly cleaved sheet of mica (V1 quality) and incubated for few minutes at room temperature. Afterwards the mica sheet was rinsed intensively with ultra-pure water (Synergy UV, Merc-Millipore) and dried with compressed air. The chromatin arrays were first diluted in buffer containing 10 mM Tris to a final DNA concentration of 30 ng/*μ*L. While EDTA is commonly added to buffers for chromatin arrays, we experienced better quality of images in Tris only buffers. In this concentration, stored in 4°C, the arrays were stable for 1-2 weeks. In the next step, the samples were further diluted in buffer containing 10 mM Tris and varying amount of NaCl to a DNA concentration of 1 ng/*μ*L. In this concentration the arrays preserve their structure for a time varying from few hours to several days, depending on the salt concentration. Subsequently, the last dilution to a DNA concentration of 125 pg/*μ*L was performed, and used for the AFM images. 160 *μ*L of this solution was deposited on poly-L-lysine covered mica and incubated for 10 to 30 minutes at room temperature just before the measurements. All buffer used for preparing arrays dilutions were centrifuged down (15 min., 20800 rcf) and filtered (cellulose acetate syringe filters, 0.25 *μ*m, VWR) in order to remove any impurities and salt crystals. Additionally, on the day of every measurement, the buffers were sonicated for at least 30 minutes in a sonication bath at a temperature of 70-80°C. Images were acquired with a Dimension FastScan Bio^™^ Atomic Force Microscope (Bruker) in tapping mode in liquid. Images at scan rate of 3-6 Hz were recorded using FASTSCAN−D probes (Bruker), having a nominal resonant frequency of 110 kHz in fluid and a nominal spring constant of 0.25 N/m. The typical amplitude setpoint was set between 200 mV and 350 mV, and the drive amplitude was between 900 mV and 1300 mV. Values used for the tapping integral gain were varying between 0.4 and 0.7. While the poly-L-lysine coating of the mica substrate increases the surface roughness (S1 Fig) this resulting volumetric contribution is negligible as compared to the imaged arrays.

### Image processing and analysis

For the quantitative analysis only images with a pixel size of 0.5 nm to 2 nm were used. All images were analysed with a custom-written (The MathWorks) software, available upon request. Images are flattened by a 3rd order polynomial fit applied both, to columns and rows, with pixel values higher than a threshold number being automatically excluded.

Recognition and reconstruction of the chromatin arrays is subsequently done in a binary representation of the flattened image, where the threshold number for the binary conversion is an image specific input with values between 0 and 1, i.e. the threshold is set relative to the range of the pixel values in the image with 0 corresponding to the lowest pixel value and 1 to the highest. Obvious artificial streaks arising from feedback errors are removed from the data. To this end, lines of 10 connected pixels in x- and y-direction with a value 1 are detected as an artificial streak in the binary image if all of these pixels have no neighbour orthogonal to the direction of the line with value 1. Every pixel of the streak is subsequently averaged in the flattened original image by the values of the two neighbouring pixels orthogonal to the direction of the streak and a new binary image is calculated.

Arrays tend to be dispersed into several sets of connected pixels; this effect occurred especially for more loosely packed structures. However, trying to resolve this by a decrease of the threshold level would artificially raise the occupied area and therefore add more noise. To overcome this problem, we developed an analysis scheme based on a graph theory [62], which enables quantification of topographies of structures even if constituted by more than one component in the binary image. To this end, every set of connected pixels is assigned a node with its size equal to the number of pixels in the respective set of connected pixels. Moreover, edges between pairs of nodes are defined using weights set to the minimal distance between the two respective sets of connected pixels in the image. However, to improve computational performance, only edges incident with one of the 20 biggest nodes were allowed, as the arrays typically contain at least one large component and many small ones. Next, all nodes smaller than a user specified minimum size (≥ 0) are erased from the graph together with all edges incident with the respective nodes, to ensure that not too much noise is added to the structures. Then a maximum distance (≥ 0) is specified, which allows a connection between two nodes only if it is smaller than the given input, i.e. a maximum distance is specified to characterize when two sets of connected pixels belong to the same structure in the image. The result is a graph with nodes greater than the minimum size and edges between two nodes closer than the maximum distance.

Single structures in the remaining graph are thus sets of nodes, which are path-connected to each other, i.e. connected sub-graphs. To find the fibres in the image, we use a breadth-first-search algorithm [63] to get the connected sub-graphs. As a structure in the image which contains more than one node is not connected in the original image, these connections have to be added for proper analysis. To interfere as little as possible, the shortest path traversing all nodes in the respective sub-graph at least once is calculated using a genetic algorithm [64] and the pixels on this path that are missing in the binary image are added to the binary image of the reconstructed array.

To quantify compaction, we use the ratio of the volume of the chromatin array as found in the analysis above to the complete area enclosed by the reconstructed array. Pixels added to the reconstruction to get path-connections in the image were not considered for the calculation of volumes, but for the calculation of the enclosed area. Holes enclosed by the structure were determined with a flood-fill algorithm [65].

Moreover, the reconstructed arrays were filtered conservatively, by discarding those that had a calculated volume equal or exceeding 200% of the theoretical volume of a 25 mer array (≈10767 nm^3^).

## Results and discussion

### Imaging of chromatin arrays in their in-solution state

**Fig 1:**
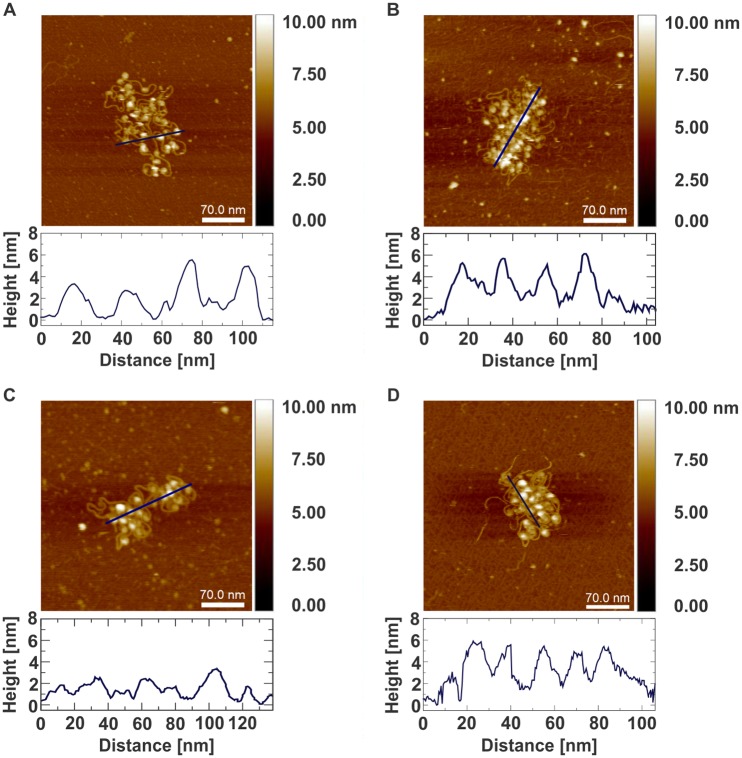
AFM imaging of chromatin arrays under different salt concentrations. Data showing arrays in A: 0 mM NaCl, B: 30 mM NaCl, C: 75 mM NaCl, D: 250 mM NaCl. Each panel displays a single array together with a height profile of a cross-section as indicated in dark blue. Pictures were chosen to best represent the average compaction level of the corresponding sodium chloride concentration.

The goal of this research was to obtain information about the in-solution compaction of nucleosomal arrays and its salt-dependence. In order to image the state of compaction we used surfaces with a high density of electrostatic charges. In particular, we use high molecular weight poly-L-lysine adsorbed to freshly cleaved mica (Materials and Methods), which has been shown to effectively attach DNA preserving its in solution characteristics [48, 49]. Experiments on free DNA molecules show that there is very limited interaction with the poly-L-lysine surface and the in-solution state of the molecule is well preserved (S2 Fig). When attaching nucleosomal arrays to poly-L-lysine surfaces, in solution characteristics are also preserved, since the rapid transfer from solution to the surfaces is happening without the freedom to rearrange upon binding. Due to this rapid binding, the in-solution structure is preserved, a procedure that resembles rapid freezing preparations for electron microscopy. This procedure yield strongly attached arrays which can be imaged using non-contact tapping mode AFM in liquid without additional chemical cross-linking (Fig 1). One should note that this attachment scheme uses DNA electrostatic interactions with the surface while the protein interfaces remain intact. The imaging was performed using a FastScan Bio^™^ Atomic Force Microscope using tapping mode. It is specifically designed for high-resolution scanning of biological samples and molecular complexes. Additionally, we used high quality, uniquely shaped AFM probes, that allowed imaging of fragile biological molecules with high scanning speed and without deforming the sample (Fig 1 and Materials and Methods).

### Salt-dependent compaction of chromatin arrays

**Fig 2:**
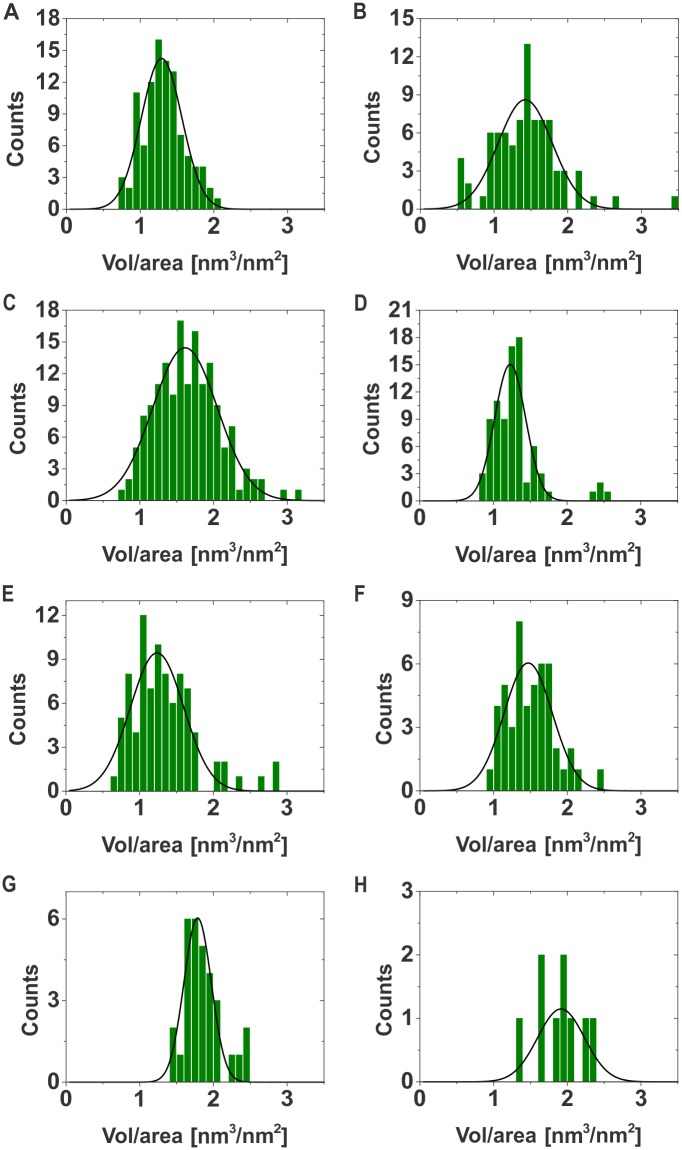
Salt-dependent chromatin array compaction. Shown are histograms (green) of chromatin array compaction, i.e. volume/area. The arrays were imaged by AFM and analysed using a graph cut algorithm to extract the volume and area occupied by each array. Plotted is the volume over area ratio. In black the Gaussian fits for each histogram are presented. A: 0 mM NaCl, B: 20 mM NaCl, C: 30 mM NaCl, D: 50 mM NaCl, E: 75 mM NaCl, F: 110 mM NaCl, G: 250 mM NaCl, H: 500 mM NaCl.

We used *in situ* AFM imaging to study the conformation of chromatin arrays as a function of the sodium chloride concentration. Different salt concentrations yielded the arrays in varying compaction states. For each salt concentration, we collected a sufficient amount of images in order to perform a statistical data analysis of array compaction (Table 1, Fig 2, Materials and Methods). In order to quantify array compaction from noisy AFM data we developed an efficient algorithm based on graph theory to compute the ratio between the volume of each single array and the surface area occupied by it (Materials and Methods).

**Table 1:** Summary of the analysed AFM data.

NaCl (mM)	Vol./area (nm^3^/nm^2^)	SE (nm^3^/nm^2^)	SD (nm^3^/nm^2^)	Analysed arrays
0	1.291	0.015	0.551	100
20	1.422	0.032	0.732	83
30	1.613	0.021	0.879	158
50	1.227	0.018	0.420	83
75	1.236	0.030	0.724	88
110	1.469	0.027	0.656	49
250	1.785	0.014	0.372	31
500	1.915	0.064	0.636	9

Table shows mean values of Gaussian distributions fitted to histograms of each analysed salt concentration, together with its standard deviation, standard error and number of analysed arrays images per subset of data. All corresponding histograms together with fitted Gaussians are presented in Fig 2.

**Fig 3:**
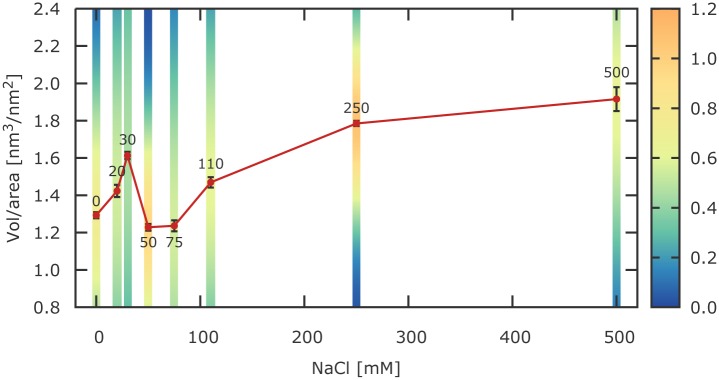
Salt-dependent compaction of the chromatin arrays. Depicted are the mean values of ratio between the volume of arrays and the surface area occupied by them, plotted against increasing NaCl concentrations (red line). Each point represents a different salt concentration and has depicted standard error (red bars). The colour coded stripes represent the normalised Gaussian distribution (area equal to 1) for each salt concentration, displaying a fairly low heterogeneity.

We recorded images for eight different salt concentrations from 0 mM to 500 mM of NaCl. For each NaCl concentration we analysed individual arrays and constructed histograms of observed compaction (Fig 2). These histograms are fitted by Gaussian functions and the results are summarised in Table 1. One should note that for all salt concentrations we observe rather broad histograms, indicating a dynamic compaction and decompaction of the arrays in solution. Previously, in salt dependent array compaction studies using analytical ultra-centrifugation, also inhomogeneous distributions have been reported [40]. When comparing the centre of the Gaussian functions for different NaCl one realized a non-monotonic behaviour of the array compaction (Fig 3 and Table 1). Analysed data shows an increase in compaction between 0 mM and 30 mM salt, followed by a drop of compaction for values between 50 mM and 75 mM NaCl. Further increase of salt, from 110 mM to 500 mM sodium chloride, yields a persistent increase in arrays’ compaction.

This quantitative observation also becomes visible when comparing the AFM images for different salt concentrations directly. In the less compacted state individual histone octamers are clearly separated by the connecting linker DNA (Fig 1C). On the other hand, when imaging highly compacted arrays (Fig 1D) free DNA is only visible at the outside of the array, while at the inside neighbouring nucleosomes are tightly associated with each other.

In order to explain this surprisingly complex salt dependence one needs to include at least three influencing energetic contributions in a physical model of array compaction. Previous studies of mononucleosomes have investigated two opposing effects on the salt-dependent stability [29]. First, increasing salt concentration reduces the ionic interactions between negatively charged DNA and positively charged histone octamers due to electrostatic screening. As a result the stability of mononucleosomes has been reported to decrease in this low to intermediate salt condition regime and also partially disassembled states have been observed where H2A/H2B dimers are partially disengaging from the rest of the structure. While a disengagement within the array is unlikely due to the inter-nucleosomal interactions, still one would expect to observe an energetic effect. Secondly, it has been shown that higher salt concentrations are favourable for the histone-histone interactions due to electrostatic screening and the screening induced hydrophobic effect, thus providing a stabilising energetic contribution for the mononucleosome. Hazan at al. used single molecule FRET studies to probe the salt dependent stability of mononucleosomes, assembled from recombinant *Xenopus laevis* histones on the ‘601’ positioning sequence [29]. They observed that the fraction of intact nucleosomes drops for increasing salt concentrations to a minimum at around 150 mM, followed by rise to maximum at about 800 mM NaCl. Even higher salt concentrations yield a reduction of the fraction of intact nucleosomes, which becomes negligible. Here, we observe a similar effect. From a maximum array compaction at 30 mM salt, compaction is decreased between 50 mM and 75 mM salt, followed by another increase of compaction for higher salt concentrations. While there is a qualitative agreement, the transition point between the two competing effects takes place at a lower salt concentration. In both studies nucleosomes were present in nano-molar concentrations, however the presence of 25 repeats on a single DNA strand, yields a much higher local concentration (estimated local concentrations of octamers are 2-10 *μ*M). Additionally, while for both array compaction as well as mononucleosome stability histone-histone interactions are important, in the former situation inter-octamer as well as intra-octamer interactions are contributing, in the latter only intra-octamer interactions are occurring. Moreover, we cannot exclude the possibility that there will be quantitative differences for human versus *Xenopus l.* histones, since *Xenopus l.* histones are known to be more stable than histones from other species [66].

While in the absence of a stable structure modelling of the salt dependent compaction is difficult, first quantitative approaches have been attempted [67]. However, the treatment depends on empirical parameters, such as the critical degree of neutralisation of the array by counter ions or the salt-dependent dissociation constant of the histones from DNA, and the contribution of monovalent salt to the self condensation of DNA is not taken into account.

A third physical property of nucleosomal arrays becomes important for the explanation of the increasing compaction from 0 mM to 30 mM salt. In the arrays, the individual nucleosomes are connected by ≈50 bps of double stranded linker DNA. It is well known that the stiffness of the DNA is salt dependent [68, 69]. This can be understood by poly-electrolyte theory due to salt dependent screening of the negatively charged DNA. While for high salt concentrations the DNA is well shielded, yielding a persistence length of about 50 nm, at salt concentrations below ≈50 mM the persistence length becomes larger, reaching ≈100 nm for vanishing salt concentrations. The increase of the persistence length for low salt is a destabilising factor for the arrays (on the scale of several tens of k_B_T), thus leading to a reduction of array compaction below 30 mM NaCl.

Interestingly, in experiments studying salt dependent array compaction using analytical ultra-centrifugation, compaction usually only increased with increasing salt concentration [40, 42]. This discrepancy arises from the fact that here, we are analysing only single arrays, whereas in the bulk experiments many arrays are known to interact with each other [33]. In fact, when we analyse AFM images where two or more arrays are forming aggregates (S3 Fig), the decrease in compaction for intermediate salt concentration begins to disappear. Moreover, competing effects as a function of NaCl concentration have been observed previously in electron microscopy experiments [33].

## Conclusion

The combination of in liquid high-resolution non-contact tapping AFM imaging and poly-L-lysine immobilisation allowed us to image chromatin arrays in solution without the use of glutaraldehyde or other cross-linking agents. Collected and analysed data revealed details about the salt-dependent compaction of the chromatin arrays. Most notably, compaction did not vary monotonically with salt concentration. We were able to describe three physical effects influencing the compaction of chromatin arrays. First, electrostatic interactions influencing the persistence length of DNA in low salt, that leads to increasing condensation of the arrays in very low salt concentrations (0 mM to 30 mM NaCl). Secondly, shielding of the DNA by salt ions, disrupts the protein- nucleic acid interactions, manifested in a drop of compaction for 50 mM and 75 mM of NaCl. This destabilising effect is countered by the stabilisation of histone-histone interactions for increasing salt, hence resulting in increasingly dense arrays structure in the salt concentrations higher than 110 mM. In our studies we did not observe any indication of defined, highly ordered forms of arrays in contrast to earlier proposals [16]. Our observations shows, that chromatin arrays have a dynamic structure, that highly depends on sodium chloride concentrations.

## Supporting information

S1 Fig Surface roughness of poly-L-lysine modified mica. The influence of surface roughness on the quantitative array compaction analysis was investigated by quantifying the observed roughness. (a) AFM image showing one individual array in a larger field of view using identical contrast as for images in the main paper. (b) Same image as in (a) but with increased contrast to highlight the surface roughness. Zoom shows magnified region as indicated. The cross-section shows the surface roughness induced noise in the image which has a negligible contribution to the overall array volume.
(PDF)

S2 Fig Exemplary AFM images of DNA on poly-L-lysine surfaces. We used DNA from the digested pUC18 plasmid to investigate the influence of the surface on the shape of the DNA molecules. Independent of salt concentration we typically observe smoothly bending polymers with estimated persistence lengths close to the in-solution value. Exemplary images for (a) 0 mM NaCl, (b) 75 mM NaCL and (c) 250 mM NaCl are shown.
(PDF)

S3 Fig Compaction of aggregated arrays. In the main analysis we introduced an upper limit for the volume of the arrays, in order to restrict the analysis to individual arrays. Here, we are presenting the analysis of the arrays whose volumes exceeded this limit. Thus, we are presenting data were two or more arrays are interacting and consequently inter-array interaction energies are also included. The histograms of observed compaction values are again fitted using Gaussian functions and the mean of theses Gaussians together with the standard error are displayed in comparison to the result of the single arrays. Remarkably the observed decrease in compaction between 50 mM and 75 mM salt is vanishing when aggregates are analysed. (a) Plot of salt dependent compaction for single (red) and multiple (blue) arrays. (b) Summary of analysed AFM data for array aggregates.
(PDF)
