# Supplementary figures and images for: Atomic force microscopy of chromatin arrays reveal non-monotonic salt dependence of array compaction in solution
Source: PLoS One. 2017 Mar 15;12(3):e0173459. doi: 10.1371/journal.pone.0173459 (PMC5351988; doi:10.1371/journal.pone.0173459)

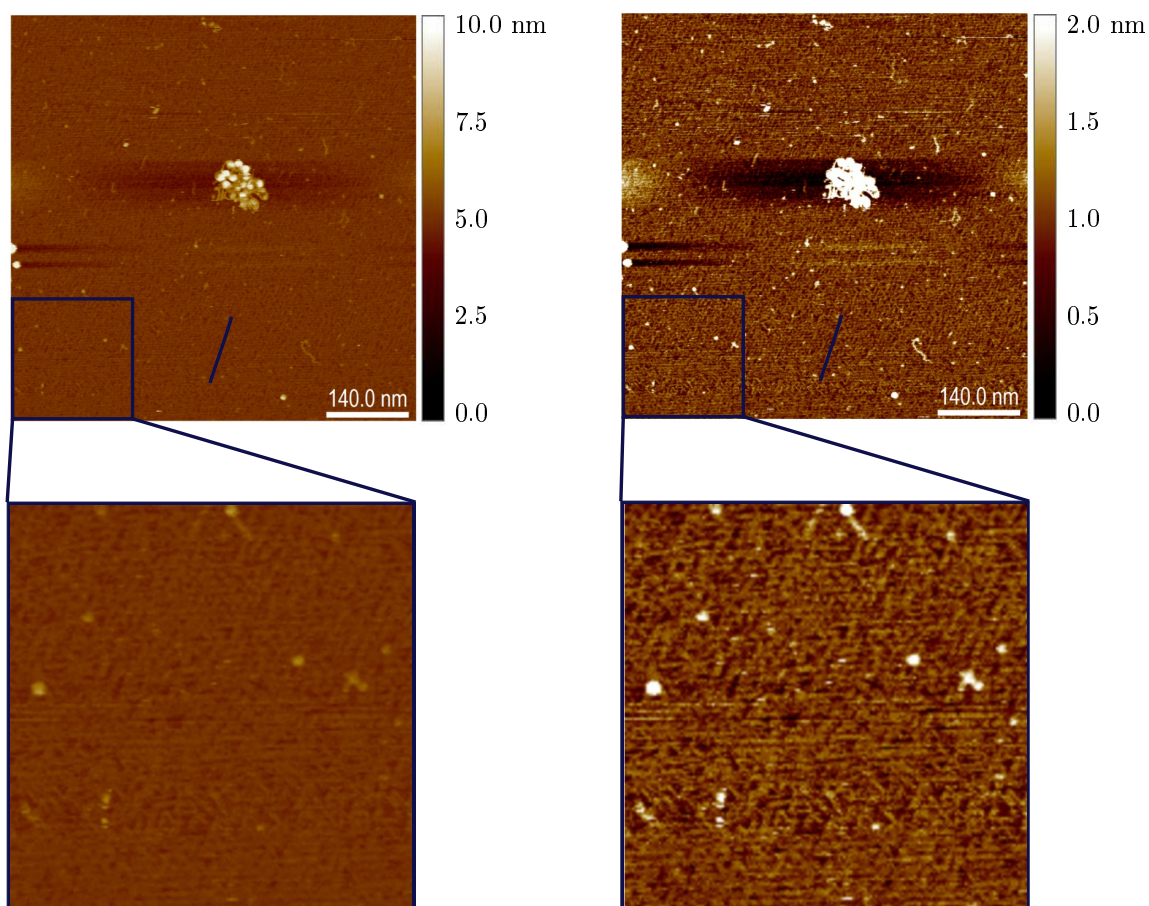

(a)

(b)

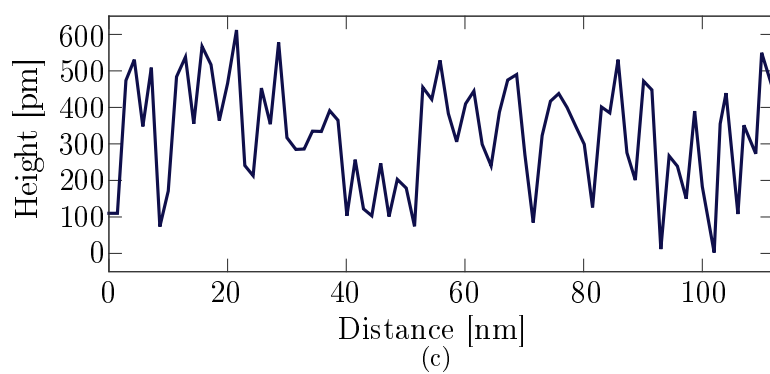

Supplement: S1 Fig — The influence of surface roughness on the quantitative array compaction analysis was investigated by quantifying the observed roughness. (a) AFM image showing one individual array in a larger field of view using identical contrast as for images in the main paper. (b) Same image as in (a) but with increased contrast to highlight the surface roughness. Zoom shows magnified region as indicated. The cross-section shows the surface roughness induced noise in the image which has a negligible contribution to the overall array volume. (PDF) [file pone.0173459.s001.pdf]

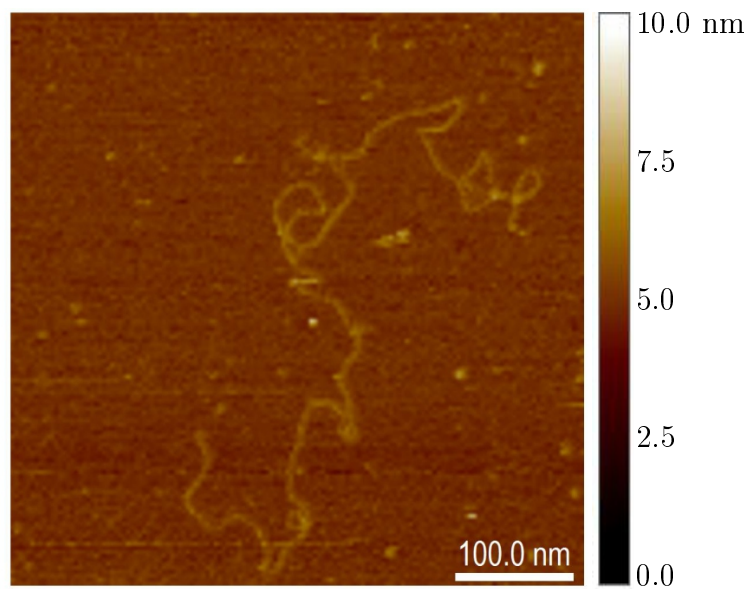

(a) 0 mM NaCl

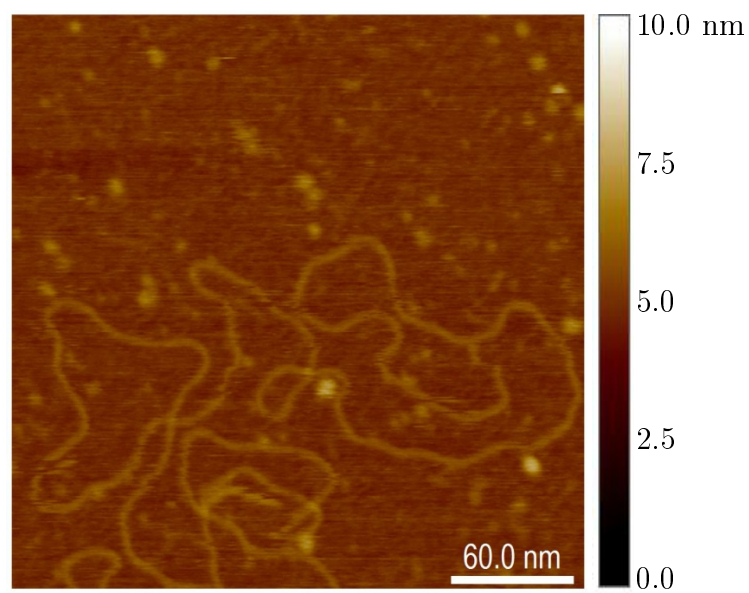

(b) 75 mM NaCl

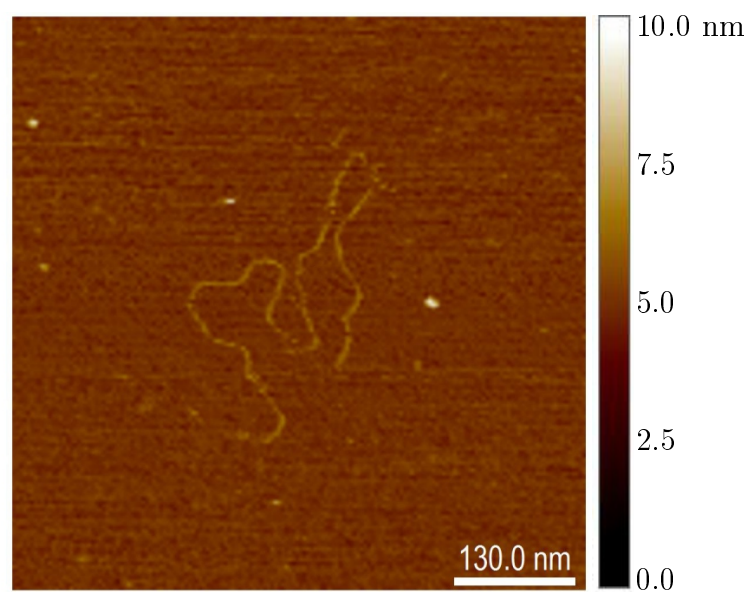

(c) 250 mM NaCl

Supplement: S2 Fig — We used DNA from the digested pUC18 plasmid to investigate the influence of the surface on the shape of the DNA molecules. Independent of salt concentration we typically observe smoothly bending polymers with estimated persistence lengths close to the in-solution value. Exemplary images for (a) 0 mM NaCl, (b) 75 mM NaCL and (c) 250 mM NaCl are shown. (PDF) [file pone.0173459.s002.pdf]

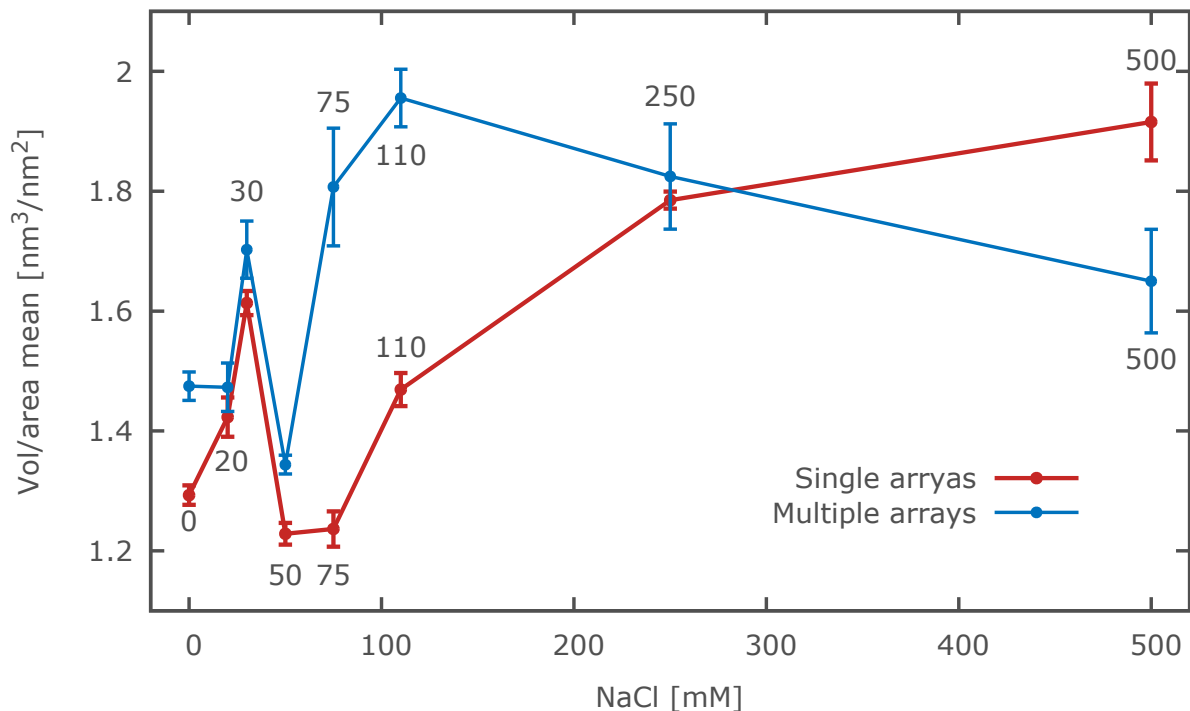

(a)

| NaCl (mM) | Vol./area ( $\text{nm}^3/\text{nm}^2$ ) | SE ( $\text{nm}^3/\text{nm}^2$ ) | SD ( $\text{nm}^3/\text{nm}^2$ ) | Analysed arrays |
|-----------|-----------------------------------------|----------------------------------|----------------------------------|-----------------|
| 0         | 1.475                                   | 0.024                            | 0.572                            | 73              |
| 20        | 1.473                                   | 0.040                            | 1.044                            | 97              |
| 30        | 1.703                                   | 0.048                            | 0.921                            | 94              |
| 50        | 1.344                                   | 0.016                            | 0.525                            | 59              |
| 75        | 1.807                                   | 0.098                            | 1.062                            | 40              |
| 110       | 1.955                                   | 0.048                            | 0.650                            | 19              |
| 250       | 1.825                                   | 0.088                            | 0.588                            | 8               |
| 500       | 1.650                                   | 0.086                            | 0.730                            | 7               |

(b)

Supplement: S3 Fig — In the main analysis we introduced an upper limit for the volume of the arrays, in order to restrict the analysis to individual arrays. Here, we are presenting the analysis of the arrays whose volumes exceeded this limit. Thus, we are presenting data were two or more arrays are interacting and consequently inter-array interaction energies are also included. The histograms of observed compaction values are again fitted using Gaussian functions and the mean of theses Gaussians together with the standard error are displayed in comparison to the result of the single arrays. Remarkably the observed decrease in compaction between 50 mM and 75 mM salt is vanishing when aggregates are analysed. (a) Plot of salt dependent compaction for single (red) and multiple (blue) arrays. (b) Summary of analysed AFM data for array aggregates. (PDF) [file pone.0173459.s003.pdf]
